# Anti-Tuberculous Vaccines and Sera

**Published:** 1906-04-28

**Authors:** 


### ANTI-TUBERCULOUS VACCINES AND SERA.

As is well known, tuberculosis may become arrested even when its advances have been considerable. Conditions arise in the human organism which are unfavourable to the growth of the bacillus, it ceases to multiply and cause fresh death of tissue, and although what has been destroyed cannot be replaced, good, if not perfect, health may be regained and the disease never make its reappearance. What these conditions are which cause arrest of the spread of the tuberculous lesions it is the aim of pathologists to discover, and to find means of aiding or of producing those conditions. Tuberculins are preparations from the tubercle bacilli, and it was hoped that they would aid the body in its fight against the disease. That they have an effect upon the subjects of tuberculosis there is not the least doubt, but as to the nature of that effect pathologists differ. Some of these views and the results of treatment by tuberculins are summarised by Dr. Fortescue-Brickdale.[1] Apparently, so far as lower animals are concerned, some tuberculins do not protect against invasion by tubercle bacilli, but against the harmful effects of the diseased products of those bacilli; while others have the power to prevent the invasion of the bacilli. In the human being there is evidence, also, that tuberculins may exert a restraining influence upon the disease, as shown by the increased power possessed by the phagocytes of the blood of destroying tubercle bacilli after the patient has been injected with tuberculin. Recent reports show also that, of 1,473 published sanatorium cases of tuberculosis treated with tuberculins, 75 per cent. have been described as cured. It is needful to remember, however, that tuberculin is of no value when the natural protective mechanism of the body is worn out—that is, in advanced cases—and is of little use when the lesions of the tubercle bacillus have become invaded by other micro-organisms, which are responsible for most of the symptoms of the disease. Tuberculins are, as has been mentioned, prepared from the tubercle bacilli themselves; anti-tuberculous sera, like other anti-toxic sera, are prepared by the injection of bacilli or of their products into animals. Although there is evidence that these sera are of some value, the number of cases treated, as yet published, is not sufficient upon which to base very definite conclusions. In addition to tuberculins and anti-tuberculous sera, Professor Behring has recently outlined the method of production of a new preparation. A bacillus is not so simple a thing as it at first appears; but though a bacillus is comparatively complicated, the methods which the body adopts to fight against its ravages are immeasurably more complicated. There must be many failures, partial or complete, before the mysteries of disease are fully elucidated, and no doubt many more failures before the forces of disease, like some other forces of nature, have to acknowledge the mastery of the mind of man.

[1] Bristol Medico-chirurgical Jour., March 1906.

